# Cutting Performance Evaluation of the Coated Tools in High-Speed Milling of AISI 4340 Steel

**DOI:** 10.3390/ma12193266

**Published:** 2019-10-07

**Authors:** Yuan Li, Guangming Zheng, Xiang Cheng, Xianhai Yang, Rufeng Xu, Huaqiang Zhang

**Affiliations:** School of Mechanical Engineering, Shandong University of Technology; 266 West Xincun Road, Zibo 255000, China; lysdut007@163.com (Y.L.);

**Keywords:** cutting performance, coated tool, wear mechanism, chip morphology, AISI 4340

## Abstract

The cutting performance of cutting tools in high-speed machining (HSM) is an important factor restricting the machined surface integrity of the workpiece. The HSM of AISI 4340 is carried out by using coated tools with TiN/TiCN/TiAlN multi-coating, TiAlN + TiN coating, TiCN + NbC coating, and AlTiN coating, respectively. The cutting performance evaluation of the coated tools is revealed by the chip morphology, cutting force, cutting temperature, and tool wear. The results show that the serration and shear slip of the chips become more clear with the cutting speed. The lower cutting force and cutting temperature are achieved by the TiN/TiCN/TiAlN multi-coated tool. The flank wear was the dominant wear form in the milling process of AISI 4340. The dominant wear mechanisms of the coated tools include the crater wear, coating chipping, adhesion, abrasion, and diffusion. In general, a TiN/TiCN/TiAlN multi-coated tool is the most suitable tool for high-speed milling of AISI 4340, due to the lower cutting force, the lower cutting temperature, and the high resistance of the element diffusion.

## 1. Introduction

The high-strength steel is widely applied in the aerospace because of its superior features. However, it is a typical difficult-to-cut material due to the high hardness, high plasticity, inferior thermal conductivity, and high toughness [1,2,3]. Therefore, it is particularly important to explore the tool materials and cutting parameters for achieving the highly-efficient machining of high-strength steel. 

Coated tools are widely used in high-speed machining (HSM) of the difficult-to-cut materials, due to their high temperature stability, high hardness, high plasticity, high toughness, excellent wear resistance, and longer tool life [4,5].

In order to study the high-speed cutting process of AISI 4340 steel, a great deal of research has been done on chip morphology, cutting force, cutting temperature, and a wear mechanism. The results show that the color of chips can reflect the cutting temperature and cutting speed when AISI 4340 steel is cut at high speed. The appearance of bright blue color chips indicates that the cutting speed is greater than 150 m/minute [6,7]. The chips are bright blue because the high cutting speeds result in high cutting temperatures, while the high cutting temperature is due to the high frictional forces between the flowing chips and the rake face [8,9]. The serrated chips are obtained when the AISI 4340 steel is cut at high speed, which can evaluate the cutting conditions. When the tooth-height of serrated chips exceeds 0.3 microns, it means that the cutting speed and the depth of cut, respectively, exceed 150 m/minute and 1.0 mm [10]. In addition, the short-broken chip is easy to gain at a high cutting speed condition [11]. The cutting parameters could be optimized by the 3D chip geometry driven analytical model [12]. As for the formation of serrated chips, it is related to crack propagation [13]. Other types of high-strength steel, such as AISI H13, also have serrated chips in the process of high-speed cutting [14]. In addition, the chips are closely related to the tool wear and the cutting force. The results show that the flow of chips can increase the probability of the coated tools’ adhesion wear [15]. Additionally, the periodicity of the serrated chip is similar to the cyclicity of the cutting force [16].

The cutting force is an important response in the HSM process of AISI 4340 steel. On the one hand, the cutting force is influenced by the type of coated tool. It is suggested that the cutting force of the CVD (chemical vapor deposition) -TiCN/Al_2_O_3_/TiN multi-coated tool is bigger than that of the PVD (physical vapor deposition) -TiAlN coated tool, due to the high friction [6]. On the other hand, the cutting parameters affect the cutting force. Based on the central composite design (CCD), the model of cutting force and cutting parameters is established. It is proven that the relationship between cutting force and cutting parameters is the quadratic polynomial [17]. Moreover, the influence of cutting speed on cutting force is more conspicuous [18]. In addition, based on the signal-to-noise ratio model, the cutting parameters are optimized by the cutting force [19].

The cutting performance of the coatings is influenced greatly by the cutting temperature [20]. In this respect, the prediction model of cutting temperature is established by the least square method [21]. This model indicates that the cutting temperature increases with the growth of cutting speed, feed per tooth, axial depth of cut, and radial depth cut. In order to predict the cutting temperature more accurately, the chip-tool interface temperature model is established [22]. In addition to establishing the model, it is also found that the thermal conductivity of the coating has an important effect on the cutting temperature. The lower thermal conductivity of Ti_0.41_Al_0.59_N coating material and cemented carbide will generate the instantaneous thermal barrier effect [23], which can effectively reduce the cutting temperature. Moreover, the TiAlN coating can improve tool life, because of the lubricating ability and anti-adhesion properties [24].

Tool wear is an important indicator for evaluating the cutting performance. Therefore, the real-time monitoring of the flank wear is realized [25], while the normal force model is established to predict the flank wear of the coated tool [26]. Furthermore, the empirical wear volume-loss equation is proposed for abrasive wear, adhesive wear, and diffusion wear. In the aspect of wear type, the dominant wear form of coated carbide tool is the flank wear in HSM of AISI 4340 steel [27]. The dominant wear mechanisms of the coated tool are adhesion, abrasion, oxidation, and diffusion in HSM of high-strength steel [6,28,29]. The cutting-edge collapses and exposed tool substrate of the TiAlN coated tool and TiCN/Al_2_O_3_/TiN multi-coated tool are common [30], and the AlTiN coated tool is more prone to chipping [31].

Based on the summary of current research on high-speed cutting of AISI 4340 steel, it was found that the main research contents are the prediction model of cutting force as well as the cutting temperature and tool wear, while the tool wear is studied. However, the most suitable coated tool for the HSM of AISI 4340 is not indicated. In the work, the TiN/TiCN/TiAlN multi-coated tool, TiAlN + TiN coated tool, TiCN + NbC coated tool, and AlTiN coated tool are used in the HSM of AISI 4340 steel. The cutting performance of the coated tools is evaluated by chip morphology, cutting force, cutting temperature, wear type, and wear mechanism in order to determine the most suitable coated tool for HSM of 4340 steel.

## 2. Materials and Methods

### 2.1. Workpiece Material

The workpiece material is AISI 4340 steel. The main chemical compositions of the workpiece are Ni 1.65%, Cr 0.72%, Mn 0.65%, C 0.40%, Mo 0.22%, Si 0.15%, P 0.01%, and S 0.01%. The tensile strength, yield strength, elongation, and reduction in area of AISI 4340 steel is 1462 MPa, 1379 MPa, at 12% and 55%, respectively [32]. The hardness of AISI 4340 steel is 42–44 HRC, which is achieved by vacuum heat treatment. The heat treatment process is completed by the manufacturer of the workpiece material. This material is made by the Dongguan Chang’an Huaming Die Steel Business Department (Dongguan, China). The length, width, and height of the workpiece are 100 mm, 100 mm, and 60 mm, respectively.

### 2.2. Cutting Tool Material

There are four types of coated tools used in the experiments. The first one is a PVD-coated carbide tool with a single coating layer made of AlTiN, with a grade number of KC522M. The second one is a PVD-coated carbide tool with a composite coating layer made of TiAlN and TiN, with grade number of PR830. The third one is a multilayer PVD-coated carbide tool consisting of three layers made by different coating material ordered from the outer to the inner layer as: TiN, TiCN, and TiAIN, respectively, with grade number of ACM300. The last one is a PVD-coated ceramic tool with a composite coating layer made of TiCN and NbC, with a grade number of TN100M. The tool hilt type of four coated tools is BT40-ER32-70. In addition, the cutter arbor types of KC522M and ACM300 is, respectively, 20A03R028A20ED10 and WEX2020E, while the cutter arbor type of PR830 and TN100M is MEC25-S20-11T. Furthermore, the cutting-edge angle of all coated tools is 90°. The cutting-edge inclination of KC522M and ACM300 are, respectively, 15° and 14°, while the cutting-edge inclination of PR830 and TN100M is 13° [33,34,35]. The geometries of coating tools have been shown in Table 1. The insert type and the diameter of cutter arbor (*d*) are shown in Table 2.

### 2.3. Experimental Design

**Figure 1:**
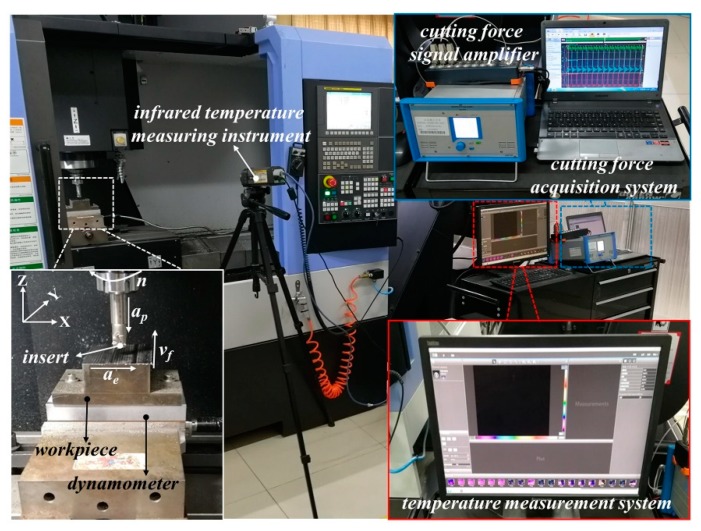
Experimental design of the high-speed milling of AISI 4340 steel.

The dry down milling tests of AISI 4340 steel are carried out by the NC milling machine center with a 3-axis, which is made in Doosan Machine Tools Co., Ltd. (Yantai, China), and model DNM-415, with a maximum speed of 12,000 r/min. To avoid the influence of the inserts, only one insert is used in the milling process. The cutting length for every cutting parameter is 300 mm. The cutting force is measured by the cutting force measurement system (Model: 9257B, Kistler, Switzerland), while the cutting temperature is measured by the thermal infrared imager (Model: A300, FLIR, Portland, OR, USA). In the process of temperature measurement, the material of the workpiece is unchanged, and the material of the coated tool is changed. To avoid the influence of the coated tool on temperature measurement, the temperature reflected by the workpiece (or chip) is selected as cutting temperature. Therefore, only the thermal diffusivity of the workpiece is set at 0.95. FLIR A300 is automatically calibrated, while the resolution and frame speed of it is 320 × 240 pixels and 30 Hz. The experimental design of the high-speed milling of AISI 4340 steel and the cutting parameters is shown in Figure 1 and Table 3, respectively. According to Table 3, each coated tool is tested in 40 different sets of cutting parameters. Furthermore, three tests were performed for each set of cutting parameters, and the average of three results is taken as experimental data. The tool wear mechanisms and the chip morphology are observed by a scanning electron microscope (SEM, Model: Quanta 250, FEI, Hillsboro, OR, USA). Chemical elements of the worn-out tools are detected by the energy dispersive spectrometer (EDS, Model: Quanta 250, FEI, Hillsboro, OR, USA).

## 3. Results and Discussion

### 3.1. Chip Morphology

**Figure 2:**
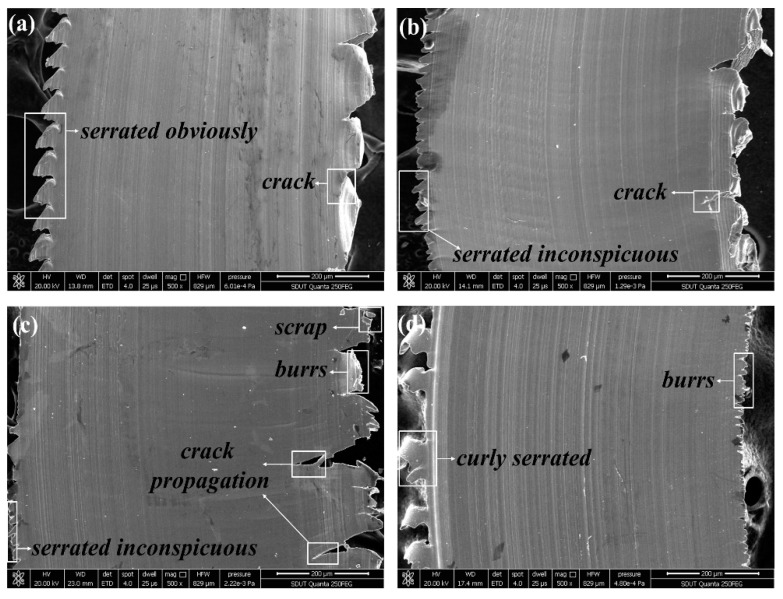
Micrographs of chip surfaces flowing from rake face at *v*_c_ = 440 m/min, *f*_z_ = 0.04 mm/z, *a*_p_ = 0.4 mm, and *a*_e_ = 4 mm, (**a**) TiN/TiCN/TiAlN multi-coating, (**b**) AlTiN coating, (**c**) TiAlN + TiN composite coating, and (**d**) TiCN + NbC composite coating.

**Figure 3:**
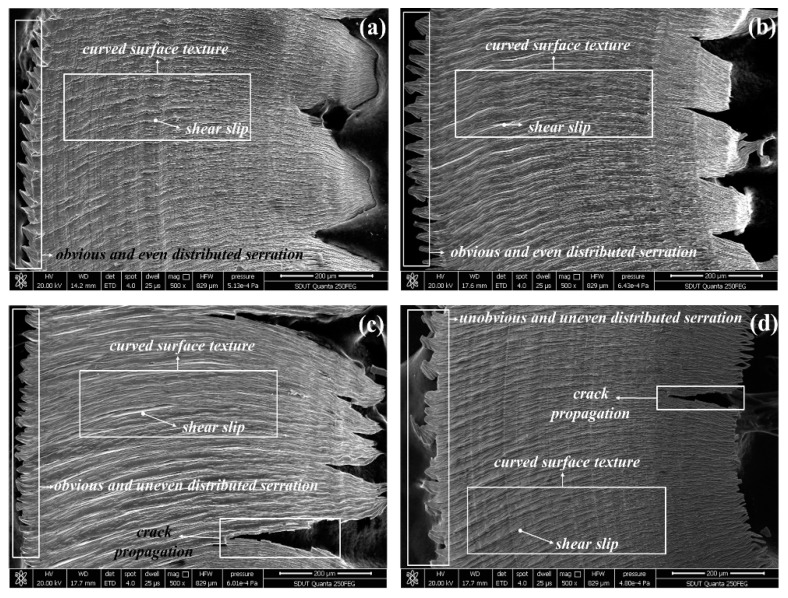
Micrographs of chip surfaces away from the rake face at *v*_c_ = 360 m/min, *f*_z_ = 0.04 mm/z, *a*_p_ = 0.4 mm, and *a*_e_ = 4 mm, (**a**) TiN/TiCN/TiAlN multi-coating, (**b**) AlTiN coating, (**c**) TiAlN +TiN composite coating, and (**d**) TiCN + NbC composite coating.

The micrographs of chip surfaces flowing from the rake face at *v*_c_ = 440 m/min, *f*_z_ = 0.04 mm/z, *a*_p_ = 0.4 mm, and *a*_e_ = 4 mm are presented in Figure 2. It is found that the crack caused by the AlTiN coated tool and the TiAlN + TiN coated tool is more serious than that caused by the TiN/TiCN/TiAlN multi-coated tool. The TiCN + NbC coated tool acquires the dense burrs chip. It is indirectly reflected that the tool wear of TiCN + NbC coated tool is enormous. As can be seen in Figure 2a, the micrograph of chip surfaces got by the TiN/TiCN/TiAlN multi-coated tool is the smoothest, which is accompanied with no clear burrs. It is suggested that the tool wear of TiN/TiCN/TiAlN multi-coating is smaller than that of the other three tools. Figure 3 shows the micrographs of chip surfaces away from the rake face at *v*_c_ = 360 m/min, *f*_z_ = 0.04 mm/z, *a*_p_ = 0.4 mm, and *a*_e_ = 4 mm, (a) TiN/TiCN/TiAlN multi-coating, (b) AlTiN coating, (c) TiAlN + TiN composite coating, and (d) TiCN + NbC composite coating. As can be seen from Figure 3, the texture of the chip surface has been curved. This proves the existence of plastic deformation. However, the difference in plastic deformation of the chip surface obtained by the four coated tools is not clear. It can be concluded that the serrated degree of chips obtained by four coated tools is different under the same cutting parameter. The formation of the serrated chips is the result of stress hardening caused by cutting force and softening caused by the cutting temperature. When the softening effect is strengthened, the stress that prevents the deformation of material will be reduced. Until the softening exceeds the stress hardening, it will cause local deformation and then produce serrated chips. In addition, the chip serration shape of the TiN/TiCN/TiAlN multi-coated tool and AlTiN coated tool was significant and uniform when compared to the TiAlN + TiN coated tool and the TiCN + NbC coated tool. This shows that stress hardening and softening of the TiN/TiCN/TiAlN multi-coated tool and the AlTiN coated tool is more stable than that of the TiAlN + TiN coated tool and the TiCN + NbC coated tool. It is also found that the crack propagation of the TiAlN + TiN coated tool and the TiCN + NbC coated tool is more serious than that of the TiN/TiCN/TiAlN multi-coated tool and the AlTiN coated tool. This indicates that the cutting force of the TiAlN + TiN coated tool and the TiCN + NbC coated tool is larger than that of the TiN/TiCN/TiAlN multi-coated tool and the AlTiN coated tool. This occurs because the severe crack propagation is caused by large cutting forces and alternating mechanical loads.

### 3.2. Cutting Force

**Figure 4:**
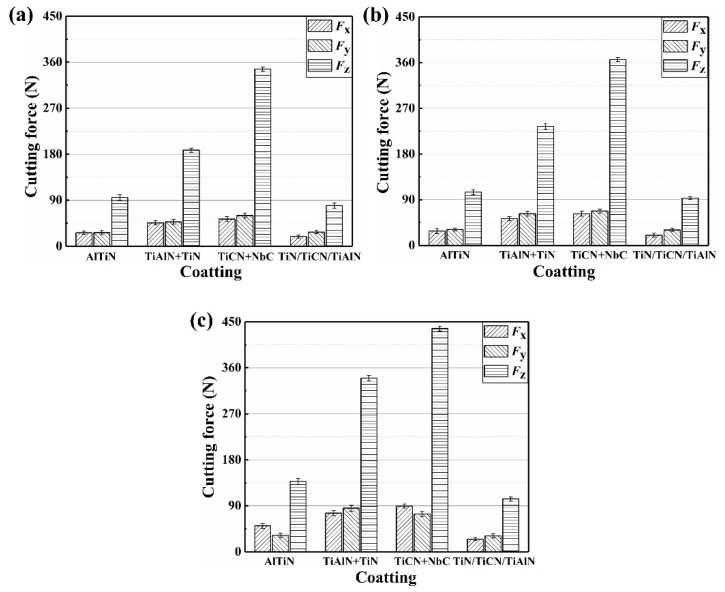
Effect of coatings on cutting force at (**a**) *v*_c_ = 280 m/min, (**b**) *v*_c_ = 360 m/min, and (**c**) *v*_c_ = 440 m/min, *f*_z_ = 0.02 mm/z, *a*_p_ = 0.2 mm, and *a*_e_ = 2 mm.

**Figure 5:**
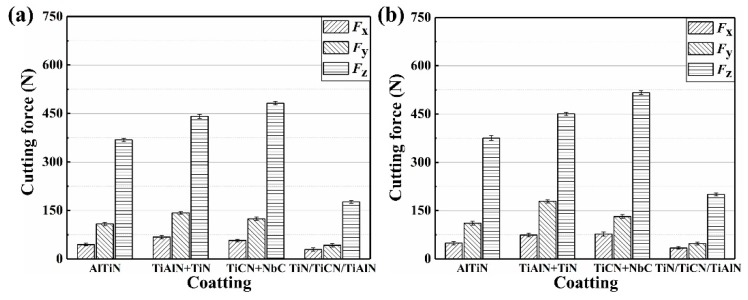

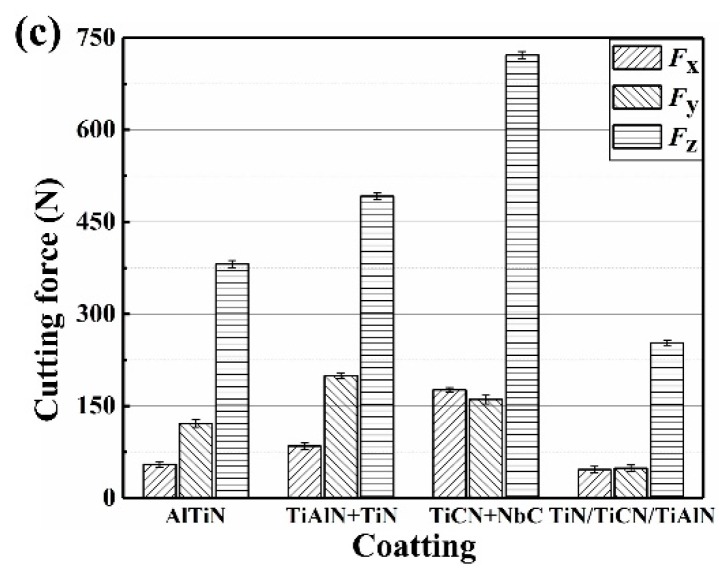
Effect of coatings on cutting force at (**a**) *v*_c_ = 280 m/min, (**b**) *v*_c_ = 360 m/min, and (**c**) *v*_c_ = 440 m/min, *f*_z_ = 0.04 mm/z, *a*_p_ = 0.4 mm, and *a*_e_ = 4 mm.

The value of cutting force (*F*_x_, *F*_y_, *F*_z_) varies with time. Therefore, the maximum force is considered to analyze the effect of coatings and cutting parameters on the cutting force. Figure 4 and Figure 5 show the effect of coatings on cutting force at different cutting parameters, from which can be seen that the *F*_z_ (axial force) is the highest component of the cutting force. It illustrates that the *F*_z_ is the main component of the cutting force. This is due to the extrusion and friction between the flank face of the coated tool and workpiece being greater than that between the rank face of the coated tool and workpiece. This is also proven by the fact that the main wear form is the flank wear. Moreover, the cutting force (*F*_x_, *F*_y_, *F*_z_) is improved with cutting speed when the *f*_z_, *a*_p_, *a*_e_, and coating material are maintaining constant, which can also be seen from Figure 4 and Figure 5. In addition, it is observed from Figure 4a and Figure 5a that the cutting force is increased with *f*_z_, *a*_p_, and *a*_e_, when the cutting speed and coating material is taking the constant value. As can be observed in Figure 4 and Figure 5, the cutting force of the TiN/TiCN/TiAlN multi-coated tool is the minimum magnitude at the same cutting parameter. Therefore, for all of that, the TiN/TiCN/TiAlN multi-coated tool is the most suitable tool for milling AISI 4340 steel to achieve the smallest cutting force.

### 3.3. Cutting Temperature

**Figure 6:**
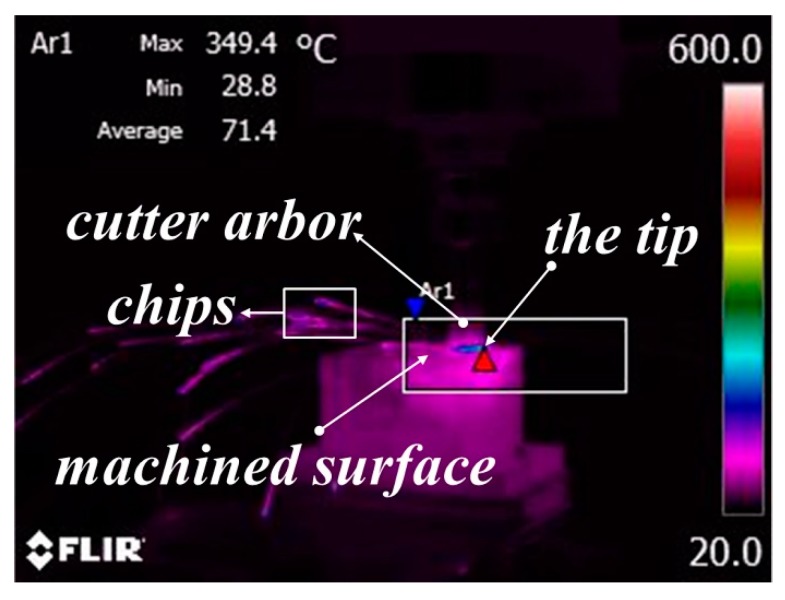
Infrared thermal image of the high-speed milling experiment.

The infrared thermal image of the high-speed milling experiment is exhibited in Figure 6. The red triangle is an automatic function of the temperature measurement system to mark the highest temperature point. In the process of temperature measurement, the temperature measurement system automatically displays a red triangle at the highest temperature in the area. The red triangle appears at the point where the tip is in contact with the workpiece, and it is concluded that the highest temperature is acquired at the contact position between the tip and the workpiece. Then, the maximum value of the cutting temperature in the machining area was investigated in the study.

**Figure 7:**
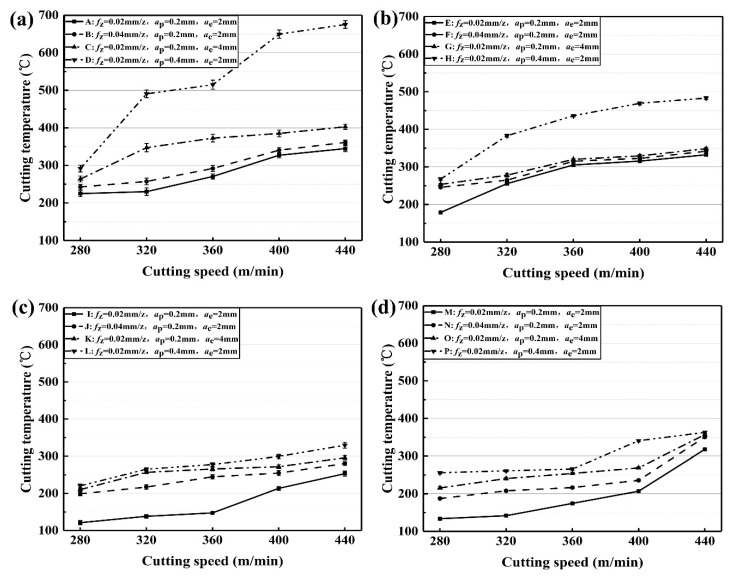
Effect of cutting parameters on cutting temperature, (**a**) TiAlN + TiN coating, (**b**) TiCN + NbC coating, (**c**) TiN/TiCN/TiAlN multi-coating, and (**d**) AlTiN coating.

Figure 7 presents the effect of cutting parameters on cutting temperature of the coated tools. The cutting temperature of TiAlN + TiN coated tool changes with the cutting parameters, as shown in Figure 7a. For the lines A and B in Figure 7a, the cutting temperature is slightly raised at *v*_c_ = 280~320 m/min and *v*_c_ = 400~440 m/min. Additionally, the growth rate of cutting temperature is improved greatly at *v*_c_ = 320~400 m/min. However, the value of line B is larger than that of line A, because of the improvement of the feed per tooth from 0.02 mm/z to 0.04 mm/z. The cutting temperature of Line C in Figure 7a increases gradually when *v*_c_ accelerates from 320 m/minute to 440 m/minute. In addition, the cutting temperature of line D is fluctuant and sharply increased with cutting speed at *a*_p_ = 0.4 mm.

The cutting temperature of TiCN + NbC coated tool changes with the cutting parameters, as shown in Figure 7b. For the lines E, F, and G, the cutting temperature slowly increases with cutting speed. However, the value of line H is clearly greater than that of lines E, F, and G. This is due to the increase of *a*_p_ from 0.2 mm to 0.4 mm. It is shown that the effect of doubling *a*_p_ on cutting temperature is greater than doubling *f*_z_ or *a*_e_ on cutting temperature under the same cutting speed for the TiCN + NbC coated tool.

The cutting temperature of the TiN/TiCN/TiAlN coated tool changes with cutting parameters, as shown in Figure 7c. The cutting temperature of Line I has a slight rise at *v*_c_ = 280~360 m/min. Afterward, it rapidly increases at *v*_c_ = 360~440 m/min. The cutting temperature of Line J is improved somewhat linearly with cutting speed. In addition, the cutting temperature of Line K rises sharply at *v*_c_ = 280~320 m/min, and increases slowly at *v*_c_ = 320~440 m/min. 

The cutting temperature of the AlTiN coated tool changes with cutting parameters, as shown in Figure 7d. For line M, the value of the cutting temperature is slightly raised at *v*_c_ = 280~320 m/minute. As *v*_c_ increases from 320 to 400 m/min, the cutting temperature rapidly increases. When the increase of *v*_c_ grows from 400 to 440 m/minute, the rise in cutting temperature grows sharply. For lines N, O, and P, the cutting temperature increases with the increase of *f*_z_, *a*_p_, or *a*_e_. In addition, the value of cutting temperature increases slightly.

In general, the cutting temperature can be improved with the increase of the cutting parameters. As the cutting parameters increase, the volume of material removed per unit time is improved. As a result, the plastic deformation of the workpiece material and chip becomes more severe. The energy generated by plastic deformation is converted into cutting heat, which leads to the rise of the temperature in the cutting area. At the same time, the influence of cutting temperature on the thermal conductivity of AISI 4340 steel is considered, because the thermal conductivity of AISI4340 steel is reduced with the increase of cutting temperature [36]. The low thermal conductivity can lead to the reduction of heat dissipation and the increase of cutting temperature.

On the other hand, at the same cutting parameter, the cutting temperature of the TiN/TiCN/TiAlN multi-coated tool is lower than that of the other coated tool, which can be seen in Figure 7. The TiN/TiCN/TiAlN coating is a multi-layer PVD-coating, and AlTiN coating is a single PVD-coating, while the TiAlN + TiN coating and TiCN + NbC coating are the composite PVD-coating. The order of the thermal conductivity is TiCN coating > TiN coating > TiAlN coating [37]. However, only the TiN/TiCN/TiAlN multi-coated tool has the middle layer of TiCN. TiCN, as the middle layer, increases the heat dissipation of the TiN/TiCN/TiAlN multi-coated tool. When the heat is transferred to the TiCN layer, the heat is rapidly dissipated due to the high thermal conductivity of TiCN. Therefore, the cutting temperature of the TiN/TiCN/TiAlN multi-coated tool is lower than that of the other coated tool.

### 3.4. Wear Type and Wear Mechanism

**Figure 8:**
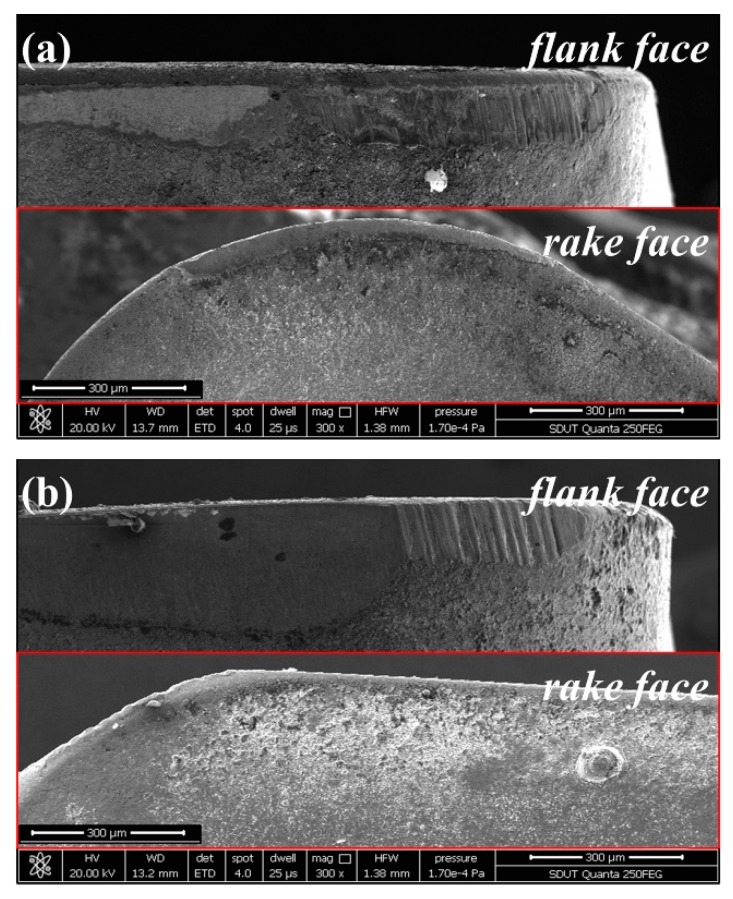
Rake wear and flank wear at *v*_c_ = 280 m/min, *f*_z_ = 0.02 mm, *a*_p_ = 0.2 mm, and *a*_e_ = 2 mm, (**a**) TiAlN + TiN coating, and (**b**) TiCN + NbC coating.

Figure 8 presents the rake wear and flank wear at *v*_c_ = 280 m/min, *f*_z_ = 0.02 mm, *a*_p_ = 0.2 mm, and *a*_e_ = 2 mm. For the TiAlN + TiN coated tool and TiCN + NbC coated tool, the wear of the rake face is less than that of the flank face. Therefore, the flank wear is a dominant wear type in high-speed milling of AISI 4340 steel.

**Figure 9:**
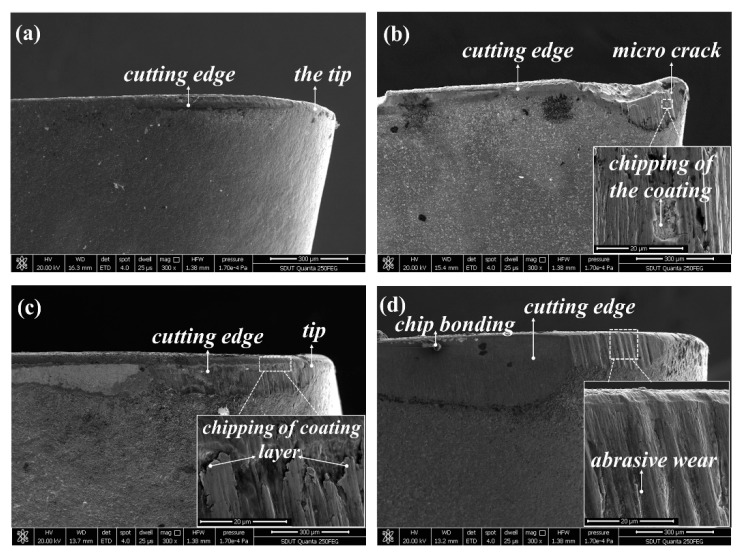
SEM micrograph of tool wear at *v*_c_ = 280 m/min, *f*_z_ = 0.02 mm, *a*_p_ = 0.2 mm, and *a*_e_ = 2 mm, (**a**) TiN/TiCN/TiAlN multi-coated tool, (**b**) AlTiN coated tool, (**c**) TiAlN + TiN coated tool, and (**d**) TiCN + NbC coated tool.

**Figure 10:**
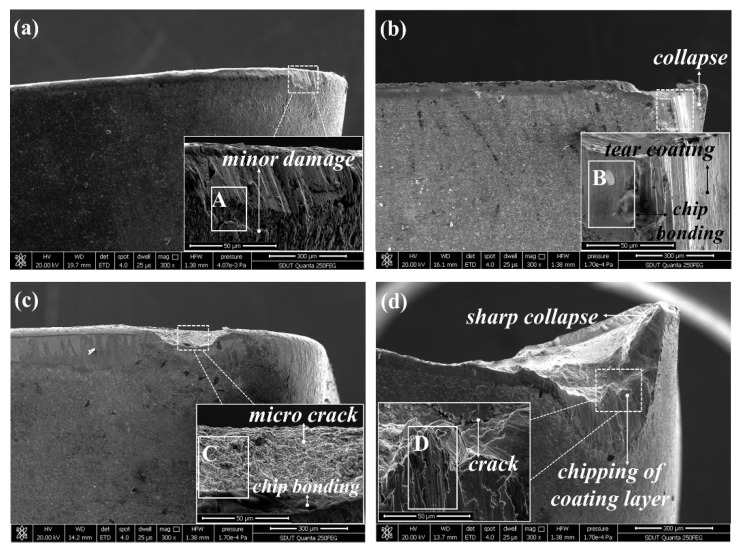
SEM micrograph of tool wear at *v*_c_ = 440 m/min, *f*_z_ = 0.04 mm, *a*_p_ = 0.4 mm, and *a*_e_ = 4 mm, (**a**) TiN/TiCN/TiAlN multi-coated tool, (**b**) AlTiN coated tool, (**c**) TiAlN + TiN coated tool, and (**d**) TiCN + NbC coated tool.

The SEM micrograph of tool wear at *v*_c_ = 280 m/min, *f*_z_ = 0.02 mm, *a*_p_ = 0.2 mm, and *a*_e_ = 2 mm is shown in Figure 9, from which can be seen that the flank wear width of the TiN/TiCN/TiAlN multi-coated tool is the minimum. The micro crack on the tip and the coating chipping are found on the worn surface of the AlTiN coated tool (Figure 9b). As can be observed in Figure 9a,c, the flank wear width got by TiAlN + TiN coated tool is more serious than that got by the TiN/TiCN/TiAlN multi-coated tool. The coating chipping of the TiAlN + TiN coated tool is also observed on the flank face (Figure 9c). For the TiCN + NbC coated tool, the flank wear width is the maximum and the chip bonding and abrasive wear is also found on the flank face, as can be illustrated in Figure 9d. Figure 10 shows the SEM micrograph of tool wear at *v*_c_ = 440 m/min, *f*_z_ = 0.04 mm, *a*_p_ = 0.4 mm, and *a*_e_ = 4 mm. For the TiN/TiCN/TiAlN multi-coated tool, the damaged coating is presented on the flank face (Figure 10a). For the AlTiN coated tool, the collapse and tear of coating are observed as the dominant wear type (Figure 10b). In addition, a typical chip bonding is observed on the flank face of the AlTiN coating. Additionally, the micro crack and chip bonding are observed on the worn surface of the TiAlN + TiN coated tool (Figure 10c). As can be exhibited in Figure 10d, the tool failure of the TiCN + NbC coated tool is mainly caused by coating chipping, crack, and sharp collapse.

As can be seen in Figure 9 and Figure 10, the sharpening, chipping, crack, and collapse is not found on the worn surface of the TiN/TiCN/TiAlN multi-coated tool. It is inferred that the wear-resistance and shock resistance of the TiN/TiCN/TiAlN multi-coated tool is better than that of the other three tools. The chipping of the coating layer and abrasive wear is not found on the flank surface of the TiN/TiCN/TiAlN multi-coated tool. The coating adhesion and cohesion between coating and the substrate of the TiN/TiCN/TiAlN multi-coated tool is compared to that of the other coated tool. In addition, the TiN/TiCN/TiAlN multi-coated tool has no serious coating damage, which can also prove the result.

**Figure 11:**
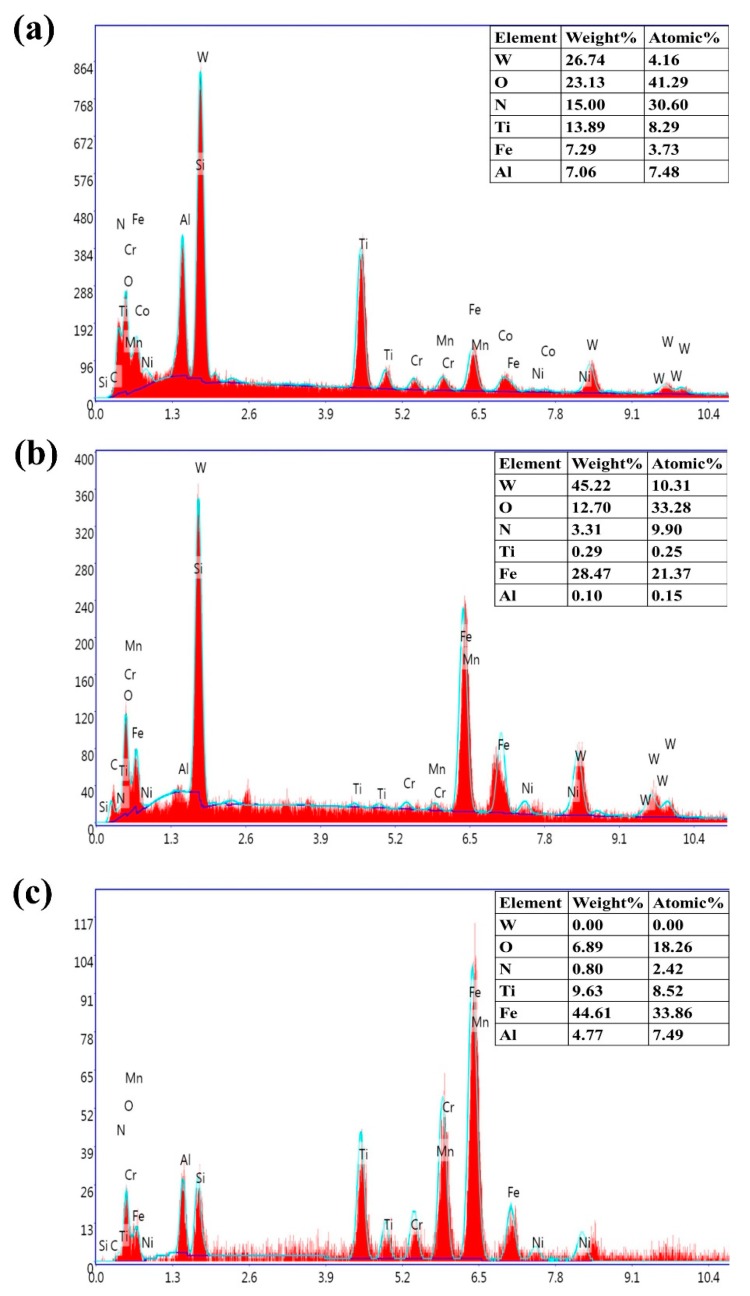

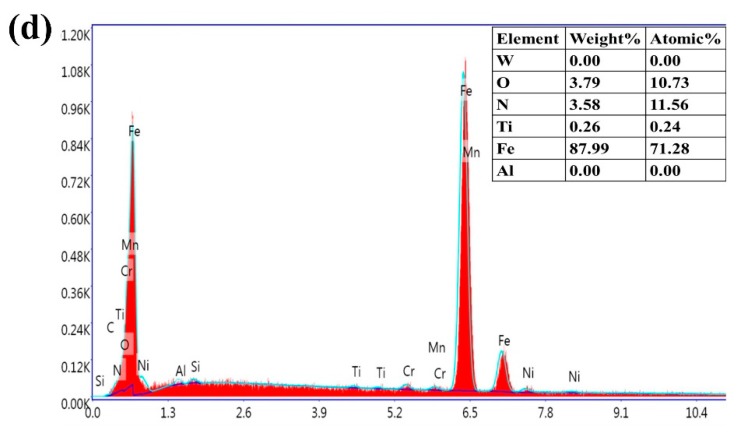
EDS analysis at *v*_c_ = 440 m/min, *f*_z_ = 0.04 mm/z, *a*_p_ = 0.4 mm, and *a*_e_ = 4 mm. (**a**) Area A in Figure 10a. (**b**) Area B in Figure 10b. (**c**) Area C in Figure 10c. (**d**) Area D in Figure 10d.

The EDS analysis at *v*_c_ = 440 m/min, *f*_z_ = 0.04 mm/z, *a*_p_ = 0.4 mm, and *a*_e_ = 4 mm is shown in Figure 11. For the TiN/TiCN/TiAlN multi-coated tool (Figure 11a), the proportion of the Ti element in the coating material is higher. It is suggested that the coating is relatively complete. This is consistent with the result of tool wear (Figure 10a). However, the proportion of the Ti element in the other three coating materials is lower, as shown in Figure 11b–d. It is proven that the coating is severely damaged. This is also shown by the tool wear, as shown in Figure 10b–d. Additionally, the oxygen element is present on the flank face of the four coated tools. It is demonstrated that the oxidative wear is an important factor leading to tool failure.

Moreover, the Fe element that diffused from the workpiece is tested on the worn surface (Figure 11). It is suggested that the adhesive layer is formed on the flank face. However, the adhesive layer is unstable, which can cause the flaking of coating on the flank face and accelerate tool wear. Thus, it is proven that the adhesion is an important cause of tool wear.

The content of the Fe element on the flank face of the TiN/TiCN/TiAlN multi-coated tool is the lowest (Figure 11a), while the flank wear width is minimal and the wear form is single (Figure 10a). The content of the Fe element on the flank face of the TiCN + NbC coated tool is the highest (Figure 11d), while the flank wear is the most serious (Figure 10d). It is concluded that the wear states of coated tools are related to the content of the Fe element. Because of the diffusion of the Fe element, the strength of the coated tool is seriously reduced, which results in sharpening, chipping, and peeling. Therefore, it could be inferred that the Fe element accelerates tool wear in high-speed milling of AISI 4340. 

In summary, the cutting performance of the TiN/TiCN/TiAlN multi-coated tool is the best among the four coated tools. First, the chip surface gained by TiN/TiCN/TiAlN multi-coated tools is the smoothest. Second, the cutting force of the TiN/TiCN/TiAlN multi-coated tool is minimal. Third, the high heat dissipation performance of the TiN/TiCN/TiAlN multi-coated tool contributed the lower cutting temperature. Lastly, the flank wear width of the TiN/TiCN/TiAlN multi-coated tool is minimal.

## 4. Conclusions

- The burrs and the smoothness’s degree of chip surfaces flowing from the rake face can reflect tool wear. Generally, the dense burrs and non-smooth chip surface are acquired in the condition of serious tool wear. The chip surfaces flowing from the rake face obtained by the TiN/TiCN/TiAlN multi-coated tool is the smoothest, and the flank wear width of the TiN/TiCN/TiAlN multi-coated tool is minimal.
- The cutting force (*F*_x_, *F*_y_, *F*_z_) is improved with the increase of the cutting parameter (*v*_c_, *f*_z_, *a*_p_, and *a*_e_). The *F*_z_ (axial force) is the main cutting force. The value of *F*_z_ is the maximum cutting force component when the coated tools are used for high-speed milling of AISI 4340 steel.
- TiCN, as the middle layer of the coated tool, can improve heat dissipation of the coated tool. This is also the reason why the TiN/TiCN/TiAlN multi-coated tool has the lowest cutting temperature. However, the heat dissipation of TiCN is reduced when TiCN and NbC act as the composite coating.
- The flank wear of the coated tools is the dominant wear type in high-speed milling of AISI 4340 steel. The chipping of coating, abrasive wear, collapse, and chipping are the dominant wear mechanisms. The flank wear of the TiN/TiCN/TiAlN multi-coated tool is lower than that of the other three tools.
- Among the four coated tools, the TiN/TiCN/TiAlN multi-coated tool is the most suitable for the high-speed milling of AISI 4340, because of the smallest cutting force and the lowest cutting temperature, which could achieve minimal flank wear.

## Figures and Tables

**Table 1:** The geometries of coating tools.

Grade No.	Length (mm)	Width (mm)	Thickness (mm)	Nose Radius (mm)
KC522M	12.05	6.75	3.75	0.40
PR830	11.00	6.70	3.80	0.80
ACM300	12.00	7.00	3.58	0.80
TN100M	11.00	6.70	3.80	0.80

**Table 2:** Insert type and the diameter of cutter arbor.

Grade No.	Insert Type	Diameter of Cutter Arbor *d* (mm)	Manufacturer
KC522M	EDCT10T304PDERLD	20	Kennametal, PA, USA
PR830	BDMT-11T308ER-JT	25	Kyocera, Japan
ACM300	AXMT 123508PEER-E	20	Sumitomo, Japan
TN100M	BDMT-11T308ER-JT	25	Kyocera, Japan

**Table 3:** Cutting parameters.

No.	Cutting Speed *v*_c_ (m/min)	Feed Per Tooth *f*_z_ (mm/z)	Axial Depth of Cut *a*_p_ (mm)	Radial Depth Cut *a*_e_ (mm)
1	280	0.02	0.2	2
2	280	0.02	0.2	4
3	280	0.02	0.4	2
4	280	0.02	0.4	4
5	280	0.04	0.2	2
6	280	0.04	0.2	4
7	280	0.04	0.4	2
8	280	0.04	0.4	4
9	320	0.02	0.2	2
10	320	0.02	0.2	4
11	320	0.02	0.4	2
12	320	0.02	0.4	4
13	320	0.04	0.2	2
14	320	0.04	0.2	4
15	320	0.04	0.4	2
16	320	0.04	0.4	4
17	360	0.02	0.2	2
18	360	0.02	0.2	4
19	360	0.02	0.4	2
20	360	0.02	0.4	4
21	360	0.04	0.2	2
22	360	0.04	0.2	4
23	360	0.04	0.4	2
24	360	0.04	0.4	4
25	400	0.02	0.2	2
26	400	0.02	0.2	4
27	400	0.02	0.4	2
28	400	0.02	0.4	4
29	400	0.04	0.2	2
30	400	0.04	0.2	4
31	400	0.04	0.4	2
32	400	0.04	0.4	4
33	440	0.02	0.2	2
34	440	0.02	0.2	4
35	440	0.02	0.4	2
36	440	0.02	0.4	4
37	440	0.04	0.2	2
38	440	0.04	0.2	4
39	440	0.04	0.4	2
40	440	0.04	0.4	4
